# [WITHDRAWN] The Impact of Policy and COVID-19 Prevention Strategies in China: A Qualitative Study

**DOI:** 10.21203/rs.3.rs-710681/v1

**Published:** 2021-07-15

**Authors:** fei fei huang, wei-ti Chen, wen xiu sun, lin zhang, hongzhou lu

**Affiliations:** Fujian Medical University; UCLA: University of California Los Angeles; Shanghai Public Health Clinical Center; Shanghai Public Health Clinical Center; Shanghai Public Health Clinical Center

## Abstract

The authors have requested that this preprint be removed from Research Square.

